# Transcriptome analyses reveal reduced hepatic lipid synthesis and accumulation in more feed efficient beef cattle

**DOI:** 10.1038/s41598-018-25605-3

**Published:** 2018-05-08

**Authors:** Robert Mukiibi, Michael Vinsky, Kate A. Keogh, Carolyn Fitzsimmons, Paul Stothard, Sinéad M. Waters, Changxi Li

**Affiliations:** 1 Department of Agricultural, Food and Nutritional Science, University of Alberta, Edmonton, Alberta T6G 2P5 Canada; 2 Lacombe Research and Development Centre, Agriculture and Agri-Food Canada, Lacombe, Alberta, T4L 1W1 Canada; 3 Animal and Bioscience Research Department, Teagasc, Grange, Dunsany, County Meath, Ireland

## Abstract

The genetic mechanisms controlling residual feed intake (RFI) in beef cattle are still largely unknown. Here we performed whole transcriptome analyses to identify differentially expressed (DE) genes and their functional roles in liver tissues between six extreme high and six extreme low RFI steers from three beef breed populations including Angus, Charolais, and Kinsella Composite (KC). On average, the next generation sequencing yielded 34 million single-end reads per sample, of which 87% were uniquely mapped to the bovine reference genome. At false discovery rate (FDR) < 0.05 and fold change (FC) > 2, 72, 41, and 175 DE genes were identified in Angus, Charolais, and KC, respectively. Most of the DE genes were breed-specific, while five genes including *TP53INP1*, *LURAP1L*, *SCD*, *LPIN1*, and *ENSBTAG00000047029* were common across the three breeds, with *TP53INP1*, *LURAP1L*, *SCD*, *and LPIN1* being downregulated in low RFI steers of all three breeds. The DE genes are mainly involved in lipid, amino acid and carbohydrate metabolism, energy production, molecular transport, small molecule biochemistry, cellular development, and cell death and survival. Furthermore, our differential gene expression results suggest reduced hepatic lipid synthesis and accumulation processes in more feed efficient beef cattle of all three studied breeds.

## Introduction

An animal’s ability to convert consumed feed into saleable meat is of central importance to the meat production industry because feeding related costs are the single largest variable expense in animal production^1–3^. As the global demand for meat products continues to increase due to population growth, and improved economic prosperity in the developed and developing world, provision of feed for meat animal production will become a potential burden on global resources including land, water, fertilizers, and labour^4–6^. In addition, environmental footprints including greenhouse gas emission associated with meat animal production have become a public concern^7^. Of meat production animals, beef cattle are the largest animals and a major contributor to environmental footprints^7^. Studies have shown that more feed efficient beef cattle not only consume less feed for the same amount of meat produced, but also have a reduced methane emission^8–10^. Therefore, decreasing production inputs through improving feed efficiency and reducing environmental footprints will be a vital step in improving the sustainability of the beef production industry.

Feed efficiency is a complex trait that can be measured using a variety of methods^5^. Residual feed intake (RFI) is one of the measurements of feed efficiency, and is defined as the difference between actual and expected feed or dry matter intake required for maintenance and growth^11^. RFI has become a more preferred measure of feed efficiency in beef cattle due to its phenotypic independence from production traits^5,12^ and moderate heritability^12,13^, which allow a reasonable response to genetic selection for more efficient animals without compromising their growth rate and mature weight.

It has been proposed that RFI is controlled by several physical, physiological and metabolic processes such as feed intake, digestion, body composition, tissue metabolism, activity and thermoregulation^14–16^. With the advancement of DNA markers and genotyping technologies, multiple candidate chromosomal regions or quantitative trait loci (QTL) that contribute to the variation of RFI in beef cattle have been identified through DNA marker based linkage and association studies^17–22^. However, the significant QTL regions and DNA variants vary largely across studies. To further identify genes associated with RFI, whole transcriptome profiling studies between beef cattle with divergent RFI phenotypes have also been performed for several tissues such as liver^23–28^, skeletal muscle^23,28,29^, adipose^28^, pituitary^28^, rumen^30^ and duodenum^28^. However, only a small proportion of the reported differentially expressed genes were shared across these studies. This discrepancy of DE genes identified across studies could be attributed to the differences in breed types, gender, tissue, and age of the animals used in the studies, as well as the differences in management and environmental conditions under which animals were raised and tested. These confounding factors hinder our understanding on genetic mechanisms that regulate RFI. Therefore, to better elucidate genetic influence on feed efficiency in beef cattle, we measured RFI on steers from three distinctive beef breeds including Angus, Charolais, and Kinsella Composite (KC) of similar age that were born, raised, and managed under the same environmental conditions, and then identified DE genes and molecular functions/processes associated with RFI within and across the breeds using whole transcriptome RNA-seq analyses of liver tissues of high and low RFI phenotype steers from each breed population.

## Results

### Difference of RFI and other performance traits between high and low-RFI groups

The averages and the t-test P-values for RFI and other performance traits are presented in Table 1. The animals used in this study had raw RFI values ranging from 1.55 to −1.096, 1.82 to −1.38, and 1.99 to −1.63 kg/day of dry matter intake for Angus, Charolais, and KC, respectively. The average RFI values of the low and high RFI steer groups were significantly different (P ≤ 1.69E-07) for all the three breed populations (Table 1). Of the RFI component traits, only DMI was significantly different between the two RFI groups for all the three populations, with low RFI or more feed efficient animals consuming significantly (P ≤ 0.01) less feed than their counterparts in the high RFI group for all the three breed populations. All the averages of growth and carcass traits as well as slaughter ages were not significantly different between the high and low RFI groups for all the studied breeds (P > 0.01).

**Table 1:** Differences of RFI and other performance traits between groups of high (n = 6) and low RFI steers (n = 6) of the three breeds, “*” indicates significant difference (P-value ≤ 0.01). RFI = residual feed intake, DMI _-_ = daily dry matter intake, ADG = average daily gain, MWT = metabolic body weight, FUREA = final ultrasound ribeye area at the end of feedlot test; FUFAT = final ultrasound backfat at the end of feedlot test; HCW = hot carcass weight; AFAT = carcass average backfat; REA = carcass ribeye area; LMY = lean meat yield; Marbling score (100–399 = trace marbling or less, 400–499 = slight marbling, 500–799 = small to moderate marbling, and 800–1199 = slightly abundant or more marbling).

	Angus			Charolais			Kinsella Composite (KC)		
Trait	L_RFI ± SE	H_RFI ± SE	P-value	L_RFI ± SE	H_RFI ± SE	P-value	L_RFI ± SE	H_RFI ± SE	P-value
RFI/kg/day	−0.84 ± 0.07	1.29 ± 0.10	9.24E-09*	−1.10 ± 0.0.08	1.15 ± 0.16	1.69E-07*	−1.29 ± 0.11	1.52 ± 0.12	1.18E-08*
DMI/kg/day	11.46 ± 0.51	13.31 ± 0.43	0.01*	10.11 ± 0.16	12.32 ± 0.16	2.21E-06*	9.21 ± 0.36	12.74 ± 0.36	3.95E-05*
ADG/kg/day	1.88 ± 0.11	1.74 ± 0.12	0.38	1.64 ± 0.04	1.67 ± 0.08	0.78	1.48 ± 0.10	1.63 ± 0.07	0.26
MWT/kg	115.58 ± 5.41	115.63 ± 2.75	0.99	120.73 ± 1.50	119.74 ± 1.79	0.68	99.7 ± 2.70	104.67 ± 2.77	0.23
FUREA/cm2	84.41 ± 1.56	80.34 ± 3.08	0.27	93.80 ± 2.27	91.99 ± 3.25	0.66	70.28 ± 2.90	74.22 ± 1.52	0.26
FUFAT/mm	9.23 ± 1.24	9.57 ± 0.68	0.73	7.08 ± 0.85	5.67 ± 0.63	0.21	8.67 ± 0.55	8.98 ± 0.45	0.67
HCW/kg	763.23 ± 44.26	753.47 ± 22.00	0.85	855.17 ± 23.18	843 ± 9.89	0.64	656.67 ± 21.52	697.33 ± 24.54	0.24
AFAT/mm	10.67 ± 1.09	12.17 ± 1.40	0.42	8.33 ± 1.11	6.67 ± 0.49	0.20	11.67 ± 1.18	10 ± 0.51	0.22
CREA/cm2	75.83 ± 2.34	74.33 ± 4.45	0.77	95.3 ± 4.44	94 ± 3.12	0.81	69.67 ± 2.54	76.33 ± 2.23	0.08
LMY/%	56.43 ± 1.18	55.2 ± 1.76	0.57	60.88 ± 1.09	61.94 ± 0.62	0.42	55.79 ± 0.87	57.81 ± 0.56	0.08
Marbling score	393.33 ± 23.47	438.33 ± 17.78	0.16	370 ± 36.79	398.33 ± 14.24	0.49	378.33 ± 20.56	378.33 ± 20.56	1.00
Slaughter age/day	488.9 ± 5.2	500.3 ± 4.4	0.12	517.3 ± 6.6	522.0 ± 5.0	0.58	445.2 ± 3.4	464.0 ± 7.1	0.04

L_RFI ± SE = trait mean values for the low RFI group ± standard error (SE); H_RFI ± SE = trait mean values for the high RFI group ± standard error (SE).

### Sequencing and alignment quality assessment

The Illumina sequencing yielded an average of 32,059,334 (SD = 2,575,908), 42,028,676 (SD = 8,852,805), and 30,259,896 (SD = 5,977,827) raw single-end sequence reads from the 12 cDNA libraries of Angus, Charolais, and KC samples, respectively. On average, the rapid run output sequencing mode produced more reads per sample (46,335,115 (SD = 5,355,272)) than the high output sequencing mode (30,931,809 (SD = 4,435,107)). The reads had an average length of 101 bp and an average Phred quality score of 36.2 ± 0.07. All reads were free of any sequencing adaptors and no read was flagged as having poor quality. On average 87% of the total sequences per sample were uniquely aligned and mapped to annotated genes in the bovine reference genome. The number of raw sequence reads, sequencing quality assessment, and alignment summary results for each sample are provided in the Supplementary excel file S1.

### Differential gene expression analysis

**Figure 1:**
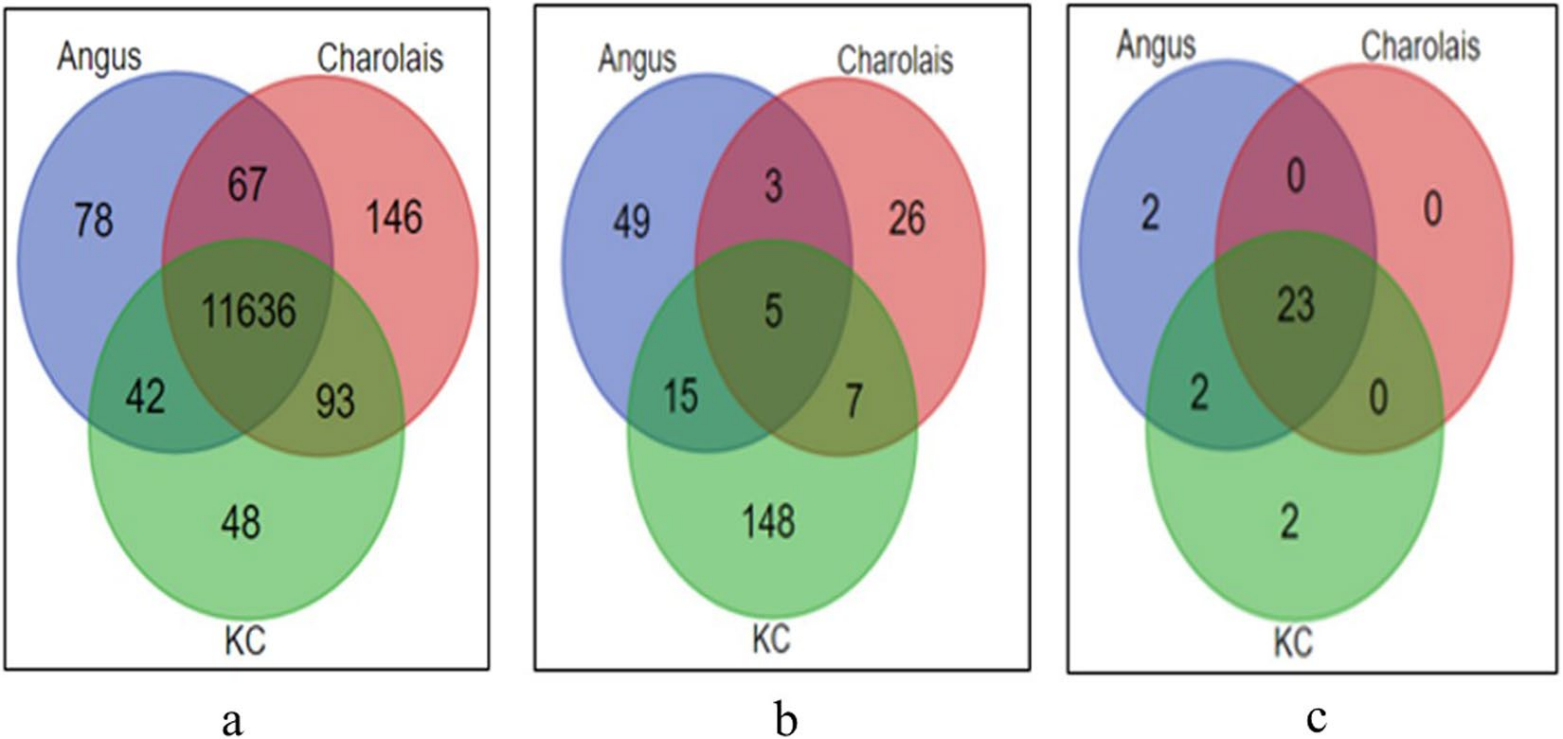
Venn diagrams showing: (**a**) overlap of expressed genes (>1CPM in ≥6 samples) in the three studied breed populations; (**b**) overlap of differentially expressed genes (DE genes) in the three studied breed populations; (**c**) overlap of biological functions enriched by DE genes identified in the three studied breed populations.

**Figure 2:**
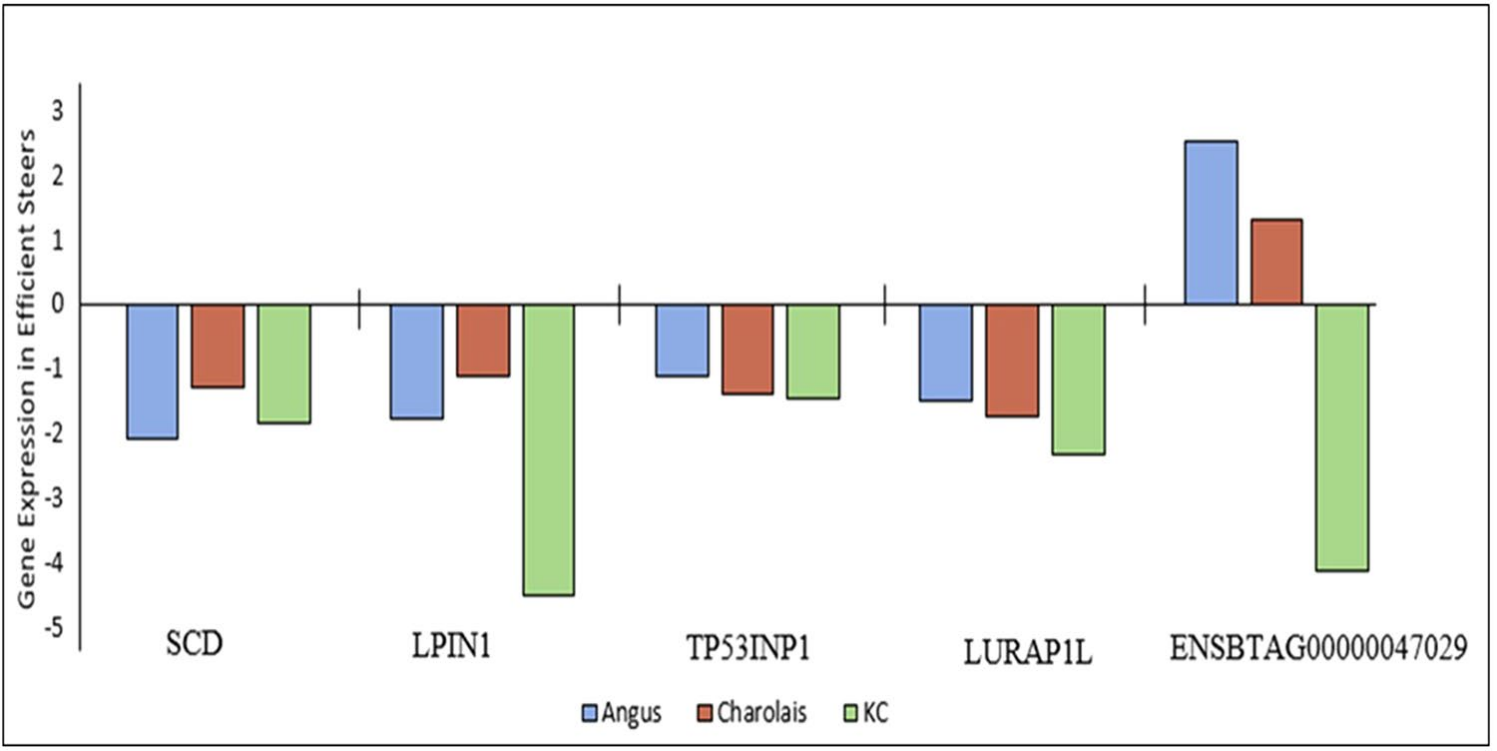
Expression profile (log_2_ (Fold-change)) in low-RFI steers of the five differentially expressed (DE) genes common across all three breeds.

After filtering out non-expressed genes, 11,823, 11,942 and 11,819 genes were found to have sufficient expression for further analyses (>1 CPM for at least half of the samples) in the liver tissues of Angus, Charolais, and KC, respectively. The majority (96.1%) of the expressed genes were common to all the three breeds as shown in Fig. 1a, hence showing a great similarity between the breeds in terms of genes expressed in the liver tissue. Of the expressed genes, 72 (46 downregulated and 26 upregulated in low-RFI steers), 41 (19 downregulated and 22 upregulated in low-RFI steers), and 175 (108 downregulated and 67 upregulated in low-RFI steers) DE genes were identified in Angus, Charolais, and KC, respectively at the significance threshold of FDR < 0.05 and FC > 2. A subset of the most significant differentially expressed genes (by FDR values) from each breed is shown in Table 2, whereas the full lists of all differentially expressed genes for each breed are provided in the Supplementary excel files S2, S3, and S4 for Angus, Charolais, and KC, respectively. When we compared DE genes across breeds, the majority of them (68.1% for Angus, 63.4% for Charolais, and 84.6% for KC) were breed specific, with only a few genes being shared between breeds (8 to 20 DE genes) or across the breeds (5) as shown in Fig. 1b. The five common DE genes across all the three breeds included *TP53INP1*, *LURAP1L*, *SCD*, *LPIN1*, and *ENSBTAG00000047029* (paralogous to *RPS23*) (Fig. 2). Four of these genes (*TP53INP1*, *LURAP1L*, *SCD* and *LPIN1*) were downregulated in all low RFI steers across the three breeds, whereas *ENSBTAG00000047029* was upregulated in low RFI steers of Angus and Charolais, but downregulated in KC low RFI steers as illustrated in Fig. 2. Between two breeds, Angus and KC shared the most unique DE genes (15), of which the majority (13) had the same expression direction in low RFI animals of the two breeds, and only two genes had a different expression direction in the efficient animals of the two breeds. Angus and Charolais shared the fewest (3) DE genes (Fig. 1b), of which *GNAZ* and *DLK1* were both downregulated in Angus, but upregulated in Charolais low RFI animals (Supplementary excel files S2 and S3).

**Table 2:** Twenty-nine of the most significant (by FDR value) differentially expressed genes in Angus, Charolais, and KC.

Angus			Charolais			Kinsella Composite (KC)		
Gene	logFC	FDR	Gene	logFC	FDR	Gene	logFC	FDR
*RPL12*	3.05	6.72E-20	*PRAP1*	−2.93	9.4E-22	*SERPINI2*	5.52	1.92E-47
*Sectm1b*	2.67	1.33E-19	*CYP2C19*	3.146	1.9E-21	*FKBP5*	−4.66	2.2E-25
*CDHR5*	−3.17	9.81E-19	*SLC13A2*	1.987	2.6E-08	*LPIN1*	−4.49	4.9E-23
*PRSS2*	−4.73	4.79E-18	*REC8*	−1.416	1.9E-05	*CYP2B6*	−3.49	9.67E-15
*HLA-DQA1*	2.99	2.55E-17	*CES1*	−1.694	3.3E-05	*CES1*	2.42	3.04E-14
*APOA4*	2.36	8.67E-14	*GPX3*	1.412	2.6E-04	*PRAP1*	−4.00	1.83E-12
*HLA-DQA2*	−2.83	3.55E-13	*LURAP1L*	−1.726	2.6E-04	*NAV2*	2.29	8.5E-11
*ECEL1*	−2.50	5.51E-11	*AK4*	1.225	2.6E-04	*AK4*	2.26	3.38E-10
*DOPEY2*	2.39	3.29E-10	*LAMB3*	−1.462	2.6E-04	*AKR1B10*	−3.98	4.73E-10
*LOC690507*	−2.77	5.79E-10	*TP53INP1*	−1.367	5.6E-04	*COL27A1*	1.94	1.17E-08
*SLC22A2*	−3.59	1.06E-09	*SLC7A5*	−1.71	07E-04	*SLC16A6*	−2.46	3.59E-08
*GIMAP4*	1.93	3.46E-09	*TMEM176B*	1.195	1.35E-03	*STS*	−2.41	4.52E-08
*SCD*	−2.07	1.40E-08	*HLA-DQB1*	−2.194	2.6E-03	*ALAS1*	−2.23	9.65E-08
*HLA-B*	−1.97	3.55E-07	*TNC*	1.233	2.71E-03	*GLCE*	−2.19	1.3E-07
*HOPX*	1.71	4.97E-07	*CXCL2*	1.474	3.37E-03	*GNMT*	−2.23	4.85E-07
*UGT2B7*	1.64	1.14E-06	*NR0B2*	−1.18	4.12E-03	*SDS*	−2.14	7.61E-07
*HLA-B*	−1.79	1.44E-06	*THEM4*	−1.239	9.12E-03	*ARG1*	−1.99	3.05E-06
*CCDC80*	−1.95	1.47E-06	*PDK4*	−1.33	0.0184	*ABHD2*	1.67	4.49E-06
*CABYR*	1.66	3.91E-06	*GPNMB*	1.155	0.0192	*NMNAT2*	−3.02	5.49E-06
*UGT2B17*	−1.78	4.86E-06	*LPIN1*	−1.118	0.0192	*PER1*	−2.03	7.37E-06
*LPIN1*	−1.77	5.94E-06	*SERPINA3*	−1.225	0.0192	*GLS2*	−1.95	7.65E-06
*SLCO4A1*	−1.87	5.94E-06	*TBATA*	1.02	0.0195	*WFDC2*	−2.02	8.16E-06
*ASCL1*	−1.71	6.26E-06	*RND1*	1.021	0.02	*MKNK1*	−1.90	1.13E-05
*IFI6*	−1.79	9.22E-06	*INMT*	1.104	0.0291	*OAT*	−2.01	1.51E-05
*RXRG*	1.42	1.06E-04	*ANXA2*	1.065	0.0309	*MFSD2A*	−2.05	1.64E-05
*FKBP5*	−1.53	2.14E-04	*SCD*	−1.275	0.0356	*MYCL*	2.03	1.88E-05
*ALAS1*	−1.54	2.47E-04	*SLC4A4*	−1.063	0.0389	*ERBB2*	1.51	4.23E-05
*TSKU*	−1.54	9.20E-04	*KLHL13*	−1.184	0.0429	*HLA-B*	−1.75	4.36E-05
*LURAP1L*	−1.49	1.26E-03	*SPNS2*	1.028	0.0466	*ASB9*	−2.80	4.80E-05

FDR = False discovery rate; logFC = log _2_(Fold-change in low RFI steers in comparison with high RFI steers).

### IPA Functional Enrichment Analysis

From the DE genes identified, 70, 37 and 169 were successfully mapped to the IPA knowledge base database for Angus, Charolais, and KC respectively. Subsequently, 27 significantly enriched biological functions (P-value < 0.05) were detected for Angus and KC, and 23 functions for Charolais. All significant biological functions and their enrichment P-values for each breed are provided in the Supplementary file S5. The majority (n = 23 or 85.2%) of the identified biological functions were common across the three studied breeds (Fig. 1c). The most significantly enriched biological functions included lipid metabolism, amino acid metabolism, carbohydrate metabolism, energy production, molecular transport, small molecule biochemistry, cellular development, and cell death and survival. Table 3 shows the DE genes involved in the top five most significantly enriched biological functions for each of the studied breeds. A full list of all biological functions identified is provided together with the list of DE genes for each breed in the Supplementary excel files S2, S3, and S4.

**Table 3:** Top five most significantly enriched biological functions within each breed and the DE genes involved within each specific function.

	Biological Function	No. of genes	Genes involved in the biological function
(**Angus**)1	Lipid metabolism	21	*ELOVL5*, *GATM*, *HP*, *LPIN1*, *ADIPOR2*, *CSF2RB*, *SLC22A2*, *CCDC80*, *ZBTB16*, *ACSS2*, *EDNRA*, *CPT1B*, *RXRG*, *APOA4*, *UGT2B17*, *SCD*, *FKBP5*, *G0S2*, *MARCO*, *PLA2G2D*, *DLK1*
2	Molecular transport	20	*ADIPOR2*, *APOA4*, *CCDC80*, *CPT1B*, *CSF2RB*, *DLK1*, *EDNRA*, *ELOVL5*, *G0S2*, *GATM*, *HP*, *LPIN1*, *MARCO*, *PLA2G2D*, *RXRG*, *SCD*, *SLC22A2*, *TP53INP1*, *UGT2B17*, *ZBTB16*
3	Small molecular biochemistry	23	*ACSS2*, *ADIPOR2*, *APOA4*, *CCDC80*, *CPT1B*, *CSF2RB*, *DLK1*, *EDNRA*, *ELOVL5*, *FKBP5*, *G0S2*, *GATM*, *HP*, *LPIN1*, *LURAP1L*, *MARCO*, *PLA2G2D*, *RXRG*, *SCD*, *SLC22A2*, *TP53INP1*, *UGT2B17*, *ZBTB16*
4	Carbohydrate metabolism	13	*SCD*, *CCDC80*, *UGT2B17*, *LPIN1*, *CSF2RB*, *PLA2G2D*, *GNAZ*, *TP53INP1*, *EDNRA*, *GATM*, *ADIPOR2*, *ELOVL5*, *APOA4*
5	Energy production	6	*SCD*, *CCDC80*, *LPIN1*, *G0S2*, *ADIPOR2*, *CPT1B*
(**Charolais**)1	Lipid metabolism	14	*ABCC4*, *AKR1C1/AKR1C2*, *ANXA2*, *CES1*, *CYP2C19*, *DLK1*, *LPIN1*, *NR0B2*, *PDK4*, *SCD*, *SLC4A4*, *SPNS2*, *THEM4*, *TNC*
2	Molecular transport	17	*ABCC4*, *AKR1C1/AKR1C2*, *ANXA2*, *CES1*, *CXCL2*, *DLK1*, *LPIN1*, *NR0B2*, *PDK4*, *SCD*, *SIRPA*, *SLC13A2*, *SLC4A4*, *SLC7A5*, *SPNS2*, *TNC*, *TP53INP1*
3	Small molecule biochemistry	21	*ABCC4*, *AK4*, *AKR1C1/AKR1C2*, *ANXA2*, *CES1*, *CYP2C19*, *DLK1*, *GPX3*, *LPIN1*, *LURAP1L*, *MIOX*, *NR0B2*, *PDK4*, *SCD*, *SLC13A2*, *SLC4A4*, *SLC7A5*, *SPNS2*, *THEM4*, *TNC*, *TP53INP1*
4	Energy production	6	*AKR1C1/AKR1C2*, *CYP2C19*, *LPIN1*, *NR0B2*, *PDK4*, *SCD*
5	Cellular development	15	*ANXA2*, *CXCL2*, *DLK1*, *GNAZ*, *GPNMB*, *LAMB3*, *LPIN1*, *NR0B2*, *PDK4*, *RND1*, *SCD*, *SIRPA*, *SLC7A5*, *TNC*, *TP53INP1*
(**KC**)1	Amino acid metabolism	22	*AASS*, *ACMSD*, *ARG1*, *ASL*, *ERBB2*, *GCH1*, *GCLC*, *GHR*, *GLS2*, *GNMT*, *GOT1*, *HAL*, *IGF1*, *IGFBP2*, *OAT*, *RXRG*, *SDS*, *SLC16A10*, *SLC22A7*, *SLC25A15*, *SLC7A2*, *TAT*
2	Small molecule biochemistry	64	*AASS*, *ABCG8*, *ACACA*, *ACMSD*, *ADA*, *AK4*, *AKR1B10*, *APOA1*, *ARG1*, *ASL*, *ASPG*, *ATP2A2*, *BAG3*, *CDKN1A*, *CES1*, *CPT1B*, *CXCL10*, *CYCS*, *CYP1A1*, *CYP2B6*, *DUSP1*, *EDNRA*, *ELOVL2*, *ERBB2*, *ERBB3*, *FGF21*,*GATA4*, *GCH1*, *GCLC*, *GHR*, *GLS2*, *GNMT*, *GOT1*, *HAL*, *HMGCR*, *IGF1*, *IGFBP2*, *INSIG1*, *LPIN1*, *MFSD2A*, *MKNK1*, *NMNAT2*, *NPC1*, *NR0B2*, *OAS1*, *OAT*, *OGDH*, *P2RY2*, *PER1*, *PNP*, *PPARGC1A*, *RBP5*, *RHOJ*, *RXRG*, *SCD*, *SDS*, *SLC16A10*, *SLC22A7*, *SLC25A15*, *SLC7A2*, *SQLE*, *STS*, *TAT*, *ZBTB16*
3	Cell death and survival	64	*ACACA*, *ADA*, *APMAP*, *APOA1*, *ARG1*, *ATP2A2*, *BAG3*, *BTG2*, *CCND1*, *CDKN1A*, *CES1*, *CXCL2*, *CXCL10*, *CYCS*, *CYP2B6*, *DDIT4*, *DUSP1*, *EDNRA*, *ERBB2*, *ERBB3*, *FGF21*, *FKBP5*, *GADD45B*, *GATA4*, *GCH1*, *GCLC*, *GHR*, *GLS2*, *GNL3*, *GNMT*, *HEYL*, *HLA-B*, *HLA-F*, *HMGCR*, *IGF1*, *IGFBP2*, *INSIG1*, *IRAK3*, *ITGA7*, *KYAT1*, *LRIG1*, *MANF*, *MKNK1*, *MOB3B*, *NMNAT2*, *NPC1*, *NR0B2*, *OAS1*, *OGDH*, *PER1*, *PIGR*, *PNP*, *PPARGC1A*, *PRAP1*, *RHOJ*, *RRS1*, *SCD*, *SERPINA3*, *TOP1*, *TP53INP1*, *TRIB2*, *UHRF1*, *USP2*, *ZBTB16*
4	Lipid metabolism	43	*ABCG8*, *ACACA*, *ADA*, *AKR1B10*, *APOA1*, *ASPG*, *ATP2A2*, *BAG3*, *CDKN1A*, *CES1*, *CPT1B*, *CXCL10*, *CYCS*, *CYP1A1*, *CYP2B6*, *DUSP1*, *EDNRA*, *ELOVL2*, *ERBB2*, *FGF21*, *GATA4*, *GHR*, *GNMT*, *GOT1*, *HMGCR*, *IGF1*, *IGFBP2*, *INSIG1*, *LPIN1*, *MFSD2A*, *MKNK1*, *NPC1*, *NR0B2*, *OGDH*, *P2RY2*, *PER1*, *PPARGC1A*, *RBP5*, *RXRG*, *SCD*, *SQLE*, *STS*, *ZBTB16*
5	Molecular transport	45	*ABCG8*, *ACACA*, *ADA*, *APOA1*, *ARG1*, *ATP2A2*, *BAG3*, *CDKN1A*, *CES1*, *CPT1B*, *CXCL10*, *CXCL2*, *CYP1A1*, *DUSP1*, *EDNRA*, *ELOVL2*, *ERBB2*, *ERBB3*, *FGF21*, *GATA4*, *GHR*, *GNMT*, *HMGCR*, *HOOK1*, *IGF1*, *INSIG1*, *LPIN1*, *MFSD2A*, *NPC1*, *NR0B2*, *P2RY2*, *PER1*, *PIGR*, *PNP*, *PPARGC1A*, *RHOJ*, *RXRG*, *SCD*, *SLC16A10*, *SLC16A6*, *SLC22A7*, *SLC25A15*, *SLC38A7*, *SLC7A2*, *ZBTB16*

**Figure 3:**
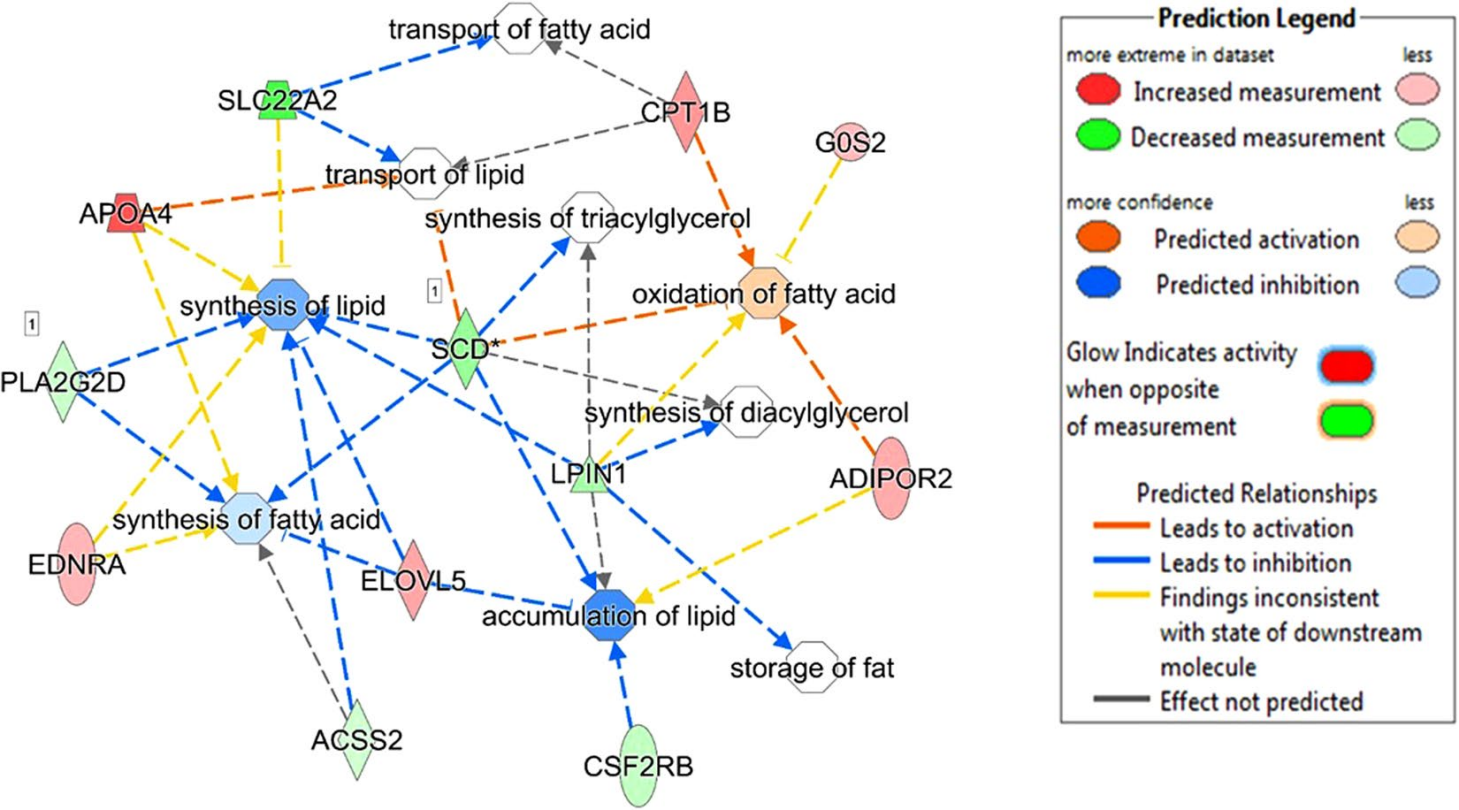
Metabolic process regulatory gene network showing differentially expressed (DE) genes involved in the different lipid metabolic processes and their predicted activation or deactivation levels in Angus low-RFI steers.

**Figure 4:**
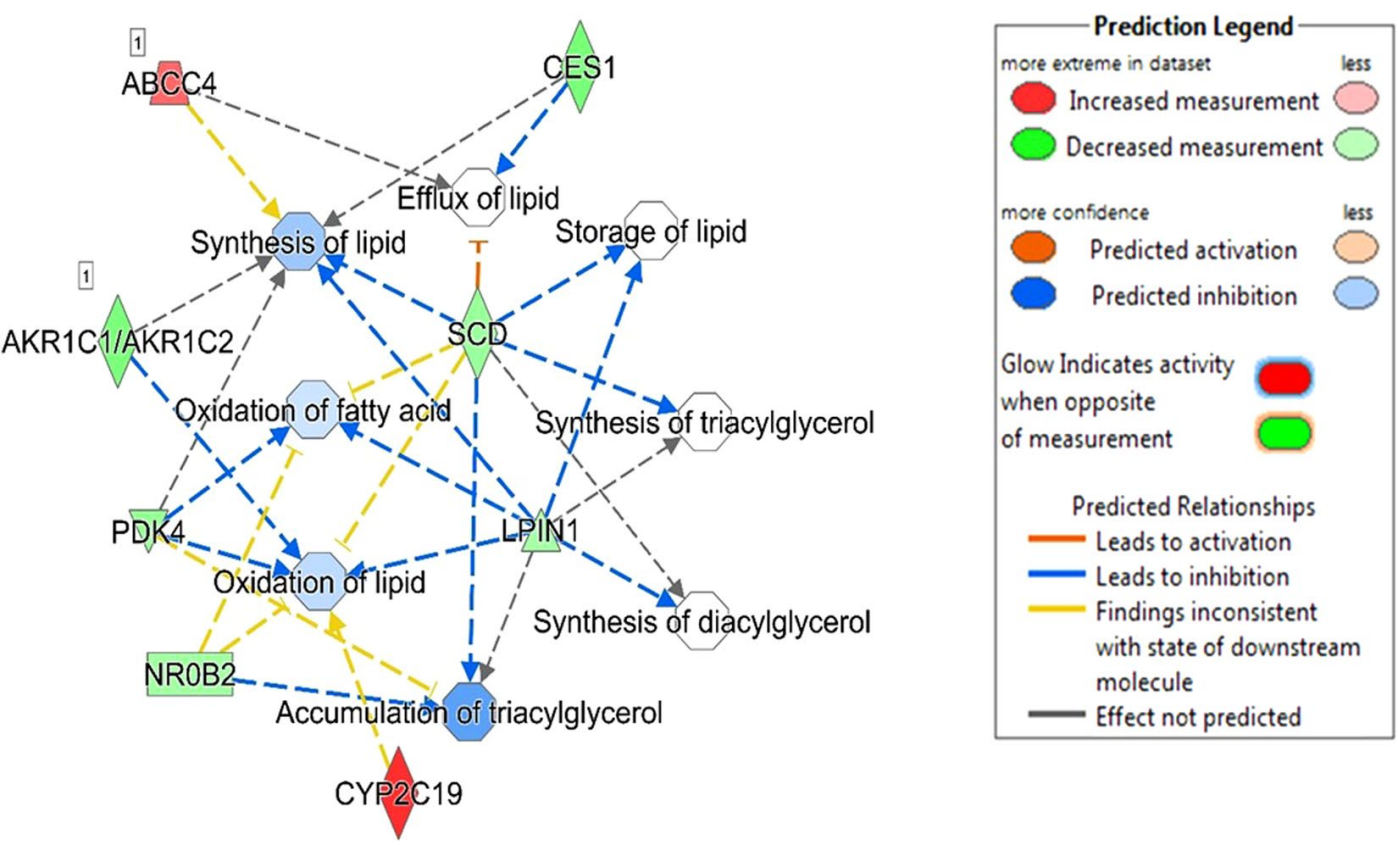
Metabolic process regulatory gene network showing differentially expressed (DE) genes involved in the different lipid metabolic processes and their predicted activation or deactivation levels in Charolais low-RFI steers.

**Figure 5:**
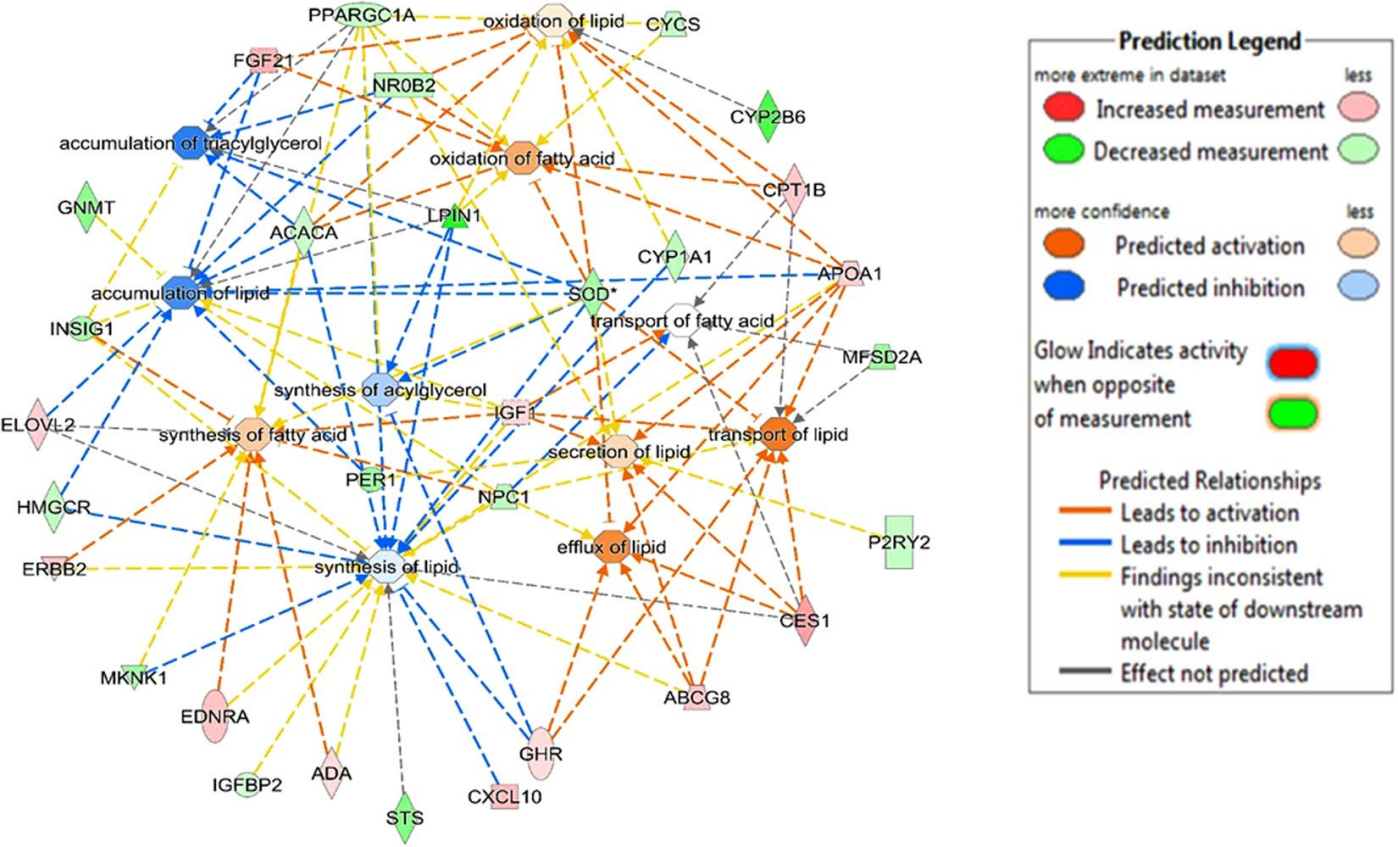
Metabolic process regulatory gene network showing differentially expressed (DE) genes involved in the different lipid metabolic processes and their predicted activation or deactivation levels in KC low-RFI steers.

Of the five shared DE genes identified in this study across the three breeds, *LPIN1* and *SCD* were involved in lipid metabolism, small molecule biochemistry, carbohydrate metabolism and energy production. *LURAP1L* was involved in small molecule biochemistry, and *TP531NP1* was involved in carbohydrate metabolism and molecular transport. Within the lipid metabolism function, further analyses of regulatory gene networks revealed several enriched fat or lipid related metabolic processes as presented in Figs 3, 4 and 5 for Angus, Charolais, and KC, respectively. Lipid synthesis was predicted to be downregulated in the liver tissues of more feed efficient animals (low-RFI steers) across all the three breeds (Figs 3, 4 and 5). Lipid accumulation was also predicted to be downregulated in Angus and KC feed efficient steers. Additionally, downregulation of accumulation of triglycerides was predicted in Charolais and KC low-RFI steers. These results indicate that more feed efficient beef cattle have reduced hepatic lipid synthesis and accumulation. However, oxidation of fatty acids was relatively upregulated in KC and Angus while downregulated in Charolais efficient steers.

## Discussion

The liver is a relatively small organ (1–2% of body mass) although metabolically it is a very active and important organ sharing 18–26% of the total body oxygen for its metabolic activities^31^. The liver is a central physiological and metabolic organ of ruminant animals. It is responsible for modulation and distribution of nutrients to peripheral tissues and organs for maintenance and production purposes such as muscle deposition in beef cattle or milk production in dairy cattle^32^. The liver is also involved in important metabolic and physiological functions relating to glucose, lipid, protein, mineral and vitamin metabolism as well as immune function, steroid hormone catabolism and detoxification of ammonia and endotoxins^33,34^. Therefore, transcriptome differences in the liver tissues between efficient and inefficient animals offer a great potential to shed some light on genes and biological functions that are involved in determining RFI in beef cattle. In the current study we employed RNA-seq to explore whole transcriptome expression differences between individuals with divergent RFI phenotypes in three beef cattle breed populations. Angus and Charolais are two distinct beef breeds with Angus being a British breed characterized by its moderate frame and early age fattening, whereas Charolais is a continental European breed with a broader frame, and later maturity and fattening^35^. KC is a composite herd composed of animals bred through crossing of multiple breeds as reported by Nkrumah *et al*.^13^. Breed composition analyses showed that the 12 KC steers used in this study had an average of 22.3% Angus and 6.7% Charolais influence along with multiple other beef breeds, indicating that KC is genetically distinct from the two pure breeds included in this study.

Our results showed that the majority of the identified DE genes related to RFI were breed or breed population specific although 96.1% of expressed genes in liver were common across the three breeds. This could be an indication that causal genes and causals mutations contributing to RFI variation in beef cattle are likely breed specific. This concurs with a low level of overlapped QTL regions of RFI across multiple breeds as reported by Saatchi and colleagues in multi-breed QTL analysis study^20^, as well as concurs with a greater discrepancy of QTL regions reported in different studies^17–19,22^. Furthermore, with respect to previous liver tissue whole transcriptomic studies in beef cattle, only 31 of the 253 DE genes identified in the current study have been previously reported in the liver tissue of beef cattle with divergent RFI phenotypes^24,27,29^, as listed in the Supplementary excel files S2, S3, and S4. It is interesting to note that of the five genes differentially expressed across all three cattle populations in our study, two genes including *Stearoyl Co-A desaturase (SCD)* and *Lipin 1* (*LPIN1*) code for key enzymes involved in lipid metabolism. Tumor protein p53 inducible protein 1 (*TP53INP1*) gene codes for a stress inducible protein (SIP) that is involved in regulation of cell death (apoptosis) and cell cycle arrest influenced by cell stressors^36^. *Leucine rich adaptor protein 1 like* (*LURAP1L*) codes for an adaptor protein reported to be involved in regulation of cell motility and migration^37^, while *ENSBTAG00000047029* codes for an uncharacterized protein and its sequence is paralogous to ribosomal protein S23 (*RPS23*) that encodes a protein that is a component of the 40 S subunit of the ribosomes (protein synthesis organelles)^38^, suggesting that these genes play key roles in altering RFI across the studied beef breeds.

Although the DE genes we identified were mainly breed specific, the enriched biological functions were greatly similar across the breeds, indicating that genes influencing RFI in beef cattle are involved in the same biological functions underlying the trait across different breeds even though the specific genes underlying RFI are different between breeds. Some of the major biological functions identified in our study included lipid metabolism, molecular transport, small molecule biochemistry, energy production, amino acid metabolism, carbohydrate metabolism, cell development, and cell death and survival. Our results showed that lipid metabolism was the most significantly enriched biological function in Angus and Charolais, and the fourth most enriched function in KC, indicating the significant biological importance of lipid metabolism in regulating RFI in beef animals. Lipid metabolism has also been previously identified as an important biological function in relation to beef cattle RFI in other hepatic transcriptome studies^24,26,28^.

Regarding lipid metabolism, our results showed that lipid synthesis (including triacylglycerol synthesis) was predicted to be downregulated in the liver tissues of low-RFI animals from all the three beef breeds (Figs 3, 4 and 5). Similarly, downregulation of genes involved in lipogenesis and steroidogenesis in both liver and fat tissue of low-RFI Yorkshire pigs has been reported by Lkhagvadorj and colleagues^39^. In a liver transcriptomic study of Nellore steers, downregulation of *fatty acid synthase* (*FASN*) was reported in steers with low residual intake and body weight gain (low-RIG)^26^, implying possible reduced fatty acid synthesis in the liver tissue of those animals. In a more recent study in Angus cattle, predicted downregulation of lipid synthesis was reported in the adipose tissue of low-RFI steers^28^. These observations suggest that feed efficient animals (not only cattle) direct consumed energy/nutrients away from lipid synthesis and probably towards protein or lean muscle synthesis. Notably, *SCD* and *LIPN1* genes identified as differentially expressed across all the three studied cattle breeds are involved in lipid synthesis. *SCD* codes for Stearoyl Co-A desaturase enzyme, a rate limiting enzyme in the biosynthesis of monounsaturated fatty acids, predominantly oleic and palmitoleic acid^40^. The synthesized fatty acids are then used as substrates for biosynthesis of other lipids such as phospholipids, triglycerides and cholesterol esters. Therefore, differential expression of this gene between feed efficient and inefficient animals may contribute to the genetic linkage/correlations between feed efficiency and carcass fatty acid composition that have been reported in beef cattle^41,42^. Differential expression of the *SCD* gene between RFI divergent beef animals has been reported in pituitary, muscle, adipose and duodenum tissues where it was also downregulated in low-RFI Angus steers^28^. *LIPN1* encodes for *Lipin-1* a phosphatidate phosphatase (PAP) enzyme, and a member of the Lipin protein family, which are mainly involved in triacylglycerol (TAG) synthesis in the glycerol phosphate pathway where they dephosphorylate phosphatidic acid to diacylglycerol^43^. Diacylglycerol is then converted to triacylglycerol by diacylglycerol transferase (*DGAT*). Triacylglycerol is a major and vital form of energy storage in adipose tissues and source of fatty acids for oxidation in both cardiac and skeletal muscles^43^. We acknowledge the fact that in ruminants such as cattle, lipogenesis or lipid synthesis predominantly occurs in the adipose tissue and a limited capacity of lipogenesis occurs in the liver^44^. This limited lipogenesis in the liver does however generate new fatty acids that are either esterified into triglycerides for storage in adipose tissue, oxidized in the liver or exported to other parts of the body as lipoproteins where they are used as a source of energy and structurally as membrane building components. Additionally, downregulation of accumulation and storage of lipids (such as triglycerides) was predicted in the low-RFI animals in all three studied breed populations. This could be another metabolic advantage that feed efficient animals have over inefficient animals. It is worth mentioning that species with limited hepatic lipogenesis like cattle also have limited potential to secrete triglycerides from the liver cells as compared to those species that use the liver as the major tissue for lipogenesis^45^. Therefore, increased hepatic lipid synthesis and accumulation as predicted in the high-RFI animals could consequently lead to increased fat accumulation in the hepatic cells of inefficient animals. Increased accumulation of fat in the liver cells may lead to the development of fatty liver^34^. Fatty liver then impairs the liver tissue’s optimal functionality of gluconeogenesis, β-oxidation, endotoxin and metabolic waste detoxification, exposing the animals to a number of metabolic stressors^34^. Interestingly, our results showed predicted upregulation of lipid secretion, transport and efflux from the hepatic cells of KC low-RFI steers which could be another mechanism of minimizing fat accumulation in those cells. In this regard, reduced liver fat synthesis and accumulation might be an adaptive metabolic or physiological advantage for feed efficient animals to maintain an optimal functioning liver tissue as compared to the inefficient animals. Although we did not perform histological evaluation of the liver tissues of the animals studied in the current study, an independent study on Nellore steers by Alexandre *et al*.^26^ through histopathological evaluation observed different liver tissue health status between the less feed efficient or high-RIG animals as compared to high efficient animals or low-RIG. In that study, they reported increased periportal liver lesions in the less feed efficient compared to high feed efficient animals, which they hypothesized was because of increased hepatic lipid biosynthesis and elevated bacterial infection in the less feed efficient animals^26^, hence revealing that hepatic tissue health could influence observed differences in feed efficiency in beef cattle.

Although phenotype records of the fat related traits (FUFAT, AFAT and marbling score) in our study did not show significant difference between the high and low RFI steer groups (Table 1), low fat accumulation or deposition in more feed efficient beef animals in different body parts has been reported by a number of studies. For example, Trejo^46^ and Nascimento *et al*.^47^ reported significantly lower internal fat content in more feed efficient beef cattle carcasses as compared to inefficient animals. Richardson and colleagues also reported lower carcass and internal fat in low-RFI Angus steers than high-RFI steers^48^. In our previous studies, it was observed that more feed efficient beef cattle tended to have less backfat and slightly less marbling^12,13^. In a more recent transcriptomic study, higher specific gravity of carcasses from feed efficient Angus steers was observed in comparison to the inefficient steers, indicating lower fat and higher lean content in the carcasses of more feed efficient animals^28^. In the same study, transcriptome analysis results predicted reduced fat synthesis and accumulation in the adipose tissue of the animals with low-RFI or more feed efficient animals^28^. Therefore, our results and the previous reports showing fat synthesis and accumulation differences between feed efficient and inefficient animals could be a result of metabolic prioritization of nutrients, especially energy. The efficient animals probably spend less energy on lipid synthesis and accumulation/deposition, which metabolically require more energy than lean tissue or protein deposition^49,50^, thus indicating that energy required to deposit fat may play a major role in determining feed efficiency in growing steers.

The liver modulates body nitrogen through several amino acid and other nitrogen compound metabolic processes, such as protein synthesis^51,52^, protein and amino acid catabolism and ureagenesis^31,32^. Indeed, our data demonstrates that amino acid metabolism was the most significantly enriched biological function in the crossbred animals with 22 DE genes involved (shown in Table 3), though only three DE genes (*SLC7A5*, *ANXA2* and *ABCC4*) and two DE genes (*GATM* and *EDNRA*) were identified as involved in amino acid metabolism in Charolais and Angus, respectively. The genes identified in KC are involved in several amino acid metabolic processes such as catabolism of amino acids (*AASS*, *ARG1*, *ASL*, *GOT1*, *HAL*, *SDS* and *TAT*), amino acid transport (*ARG1*, *IGF1*, *SLC16A10*, *SLC22A7*, *SLC25A15* and *SLC7A2*) and the urea cycle (*ARG1* and *ASL*). Even though we could not obtain activation/deactivation prediction scores for the identified processes from IPA because of low DE gene numbers, the majority of the DE genes identified in these processes were downregulated in low-RFI steers. For example, of the seven genes involved in amino acid catabolism, six (*ARG1*, *ASL*, *GOT1*, *HAL*, *SDS* and *TAT*) were downregulated in low-RFI animals, and this suggests reduced protein and amino acid breakdown in the feed efficient animals. *Argininosuccinate lyase* (*ASL*) and *arginase* (*ARG1*) are key enzymes in ureagenesis, where *Argininosuccinate lyase* catalyzes conversion of argininosuccinate to arginine, and arginase catalyzes conversion of arginine to urea and ornithine^53^. Hence, downregulation of these genes could be an indication of reduced amino acid catabolism and/or reduced synthesis of urea in the liver. Lower levels of blood urea concentration have been reported in low-RFI steers as compared to high-RFI beef cattle by Richardson *et al*.^54^ and Fitzsimons *et al*.^10^, suggesting that amino acid metabolism also plays a considerable role in regulating RFI of beef cattle.

Carbohydrate metabolism was another interesting enriched biological function in our study with 13 DE genes involved in Angus (genes shown in Table 3), 10 in Charolais, and 31 in the crossbred KC population (genes of both populations shown in Supplementary files S3 and S4). Association between RFI variation and carbohydrate metabolism has been previously reported in a liver whole transcriptome study between efficient and inefficient Angus steers^24^. More interestingly, some of the DE genes we identified are involved in gluconeogenesis, and these included *ADIPORA*, *GATM* and *SCD* for Angus, *NROB2* and *SCD* for Charolais, and *DUSP1*, *FGF21*, *GNMT*, *NROB2*, *PPARGC1A*, *SCD*, *SDS* and *TAT* for KC. Carbohydrates are a very important nutrient to an animal as they provide more than half of the total energy needed by an animal for maintenance, growth and production (muscle deposition in beef cattle)^55^. Furthermore, glucose is the main source of metabolic energy in the body, however, in ruminants most of the carbohydrates (cellulose and starch) are fermented by rumen microbes into volatile fatty acids (VFAs) which are absorbed into the blood stream and transported to the liver^55^, where VFAs are utilized for biosynthesis of several organic molecules including carbohydrates. Therefore, differential expression of genes involved in carbohydrate metabolism between inefficient and efficient animals may reflect the difference in catabolic or anabolic efficiency difference in carbohydrate synthesis and utilization by these animals.

## Conclusions

We investigated differential gene expression through RNA-seq analyses in the liver tissues of steers with divergent feed efficiency phenotypes from two beef pure breeds and a composite breed population that were born, raised and managed under the same environments, and with a similar age. We identified a total of 253 unique genes associated with RFI in the three Canadian beef cattle breeds, of which five DE genes were shared across all three breeds. The study showed a great similarity in the biological functions associated with RFI across the three breeds, with lipid metabolism, amino acid metabolism, carbohydrate metabolism, molecular transport, energy production, small molecule biochemistry, cell death and survival, and cellular development being the major functions we identified. Our results further suggest reduced hepatic lipid synthesis and fat accumulation in more feed efficient beef cattle across all the studied breeds, which may be an indication of energy prioritization away from lipid deposition and towards lean growth or maintaining better health or function of liver tissue. However, most of DE genes identified in this study were breed specific, which indicates that most causative genetic mutations contributing to RFI variation are likely not the same across beef breeds or expressed differently in different breeds. Further studies including blood tissue whole metabolome profiling, liver lipid biosynthesis and accumulation evaluation, and transcriptome analyses from multiple tissues at various developmental stages would help generate a better understanding of the genetic influence and would contribute to identification of causative mutations for RFI in beef cattle, especially when different beef breeds are examined.

## Materials and Methods

### Animal populations and management

All animals used in this study were managed according to the guidelines established by the Canadian Council of Animal Care^56^ and the experiment procedures were approved by the University of Alberta Livestock Animal Care and Use Committee (AUP00000777). Beef steers from three beef cattle herds including purebred Angus, purebred Charolais, and Kinsella Composite (KC) were used in this study. The three beef cattle herds were located and managed alike at the Roy Berg Kinsella Ranch, University of Alberta, Canada. These cattle herd populations were described previously^12,19^. Briefly, the purebred Angus and Charolais cows were bred by artificial insemination (AI) and natural service bulls with their pedigree information maintained by the Canadian Angus or Charolais Associations, respectively. The KC herd was produced from crosses between Angus, Charolais, or Alberta Hybrid bulls and the University of Alberta’s hybrid dam line that was generated by crossing composite cattle lines of multiple beef breeds as described by Goonewardene *et al*.^57^. The animals used in this study were born between April to May of 2014 and were weaned at approximately six months of age. They were then fed a background diet composed of 80% barley silage, 17% barley grain, and 3% rumensin pellet supplement, and then a transition diet with gradually decreasing barley silage and increasing barley grain proportions for 3 weeks prior to the finishing diet of 75% barley grain, 20% barley silage, and 5% rumensin pellet supplement (as fed basis).

### Growsafe feedlot test and residual feed intake calculation

In 2015, 50 Angus, 48 Charolais, and 158 KC steers were measured for individual feed intake between April to August using the GrowSafe system® (GrowSafe Systems Ltd., Airdrie, Alberta, Canada), and were fed a finishing diet during the feed intake test. Details of individual animals’ daily feed intake data collection using the GrowSafe automated system was described previously by Mao *et al*.^12^. Briefly, daily dry matter intake (DMI) of each steer was calculated as the average of daily feed intakes over the test period (70 to 73 days), standardized to 12 MJ ME per kg dry matter based on the energy content of the diet. Initial body weight and average daily gain (ADG) for each animal were obtained from a linear regression of serial body weight (BW) measurements that were recorded on two consecutive days at the beginning, at approximately 14 day intervals during the feedlot test, and on two consecutive days at the end of test. Metabolic body weight (MWT) was calculated as midpoint BW^0.75^, where midpoint BW was computed as the sum of initial BW of the animal and the product of its ADG multiplied by half the number of days under the feedlot test. The expected DMI for each animal was predicted using the regression intercept and regression coefficients of ADG and MWT on actual standardized daily DMI, and RFI was computed as the difference between the actual standardized daily DMI and the expected DMI as proposed by Koch *et al*.^11^.

### Liver tissue collection

Animals were slaughtered at Agriculture and Agri-Food Canada (AAFC) Lacombe Research Centre (Lacombe, AB) between July and September of 2015. Steers were targeted for slaughter at a backfat thickness of 8–10 mm between the 12th and 13th ribs as measured by ultrasound using an Aloka 500 V diagnostic realtime ultrasound machine with a 17 cm 3.5 Mhz linear array transducer (Overseas Monitor Corporation Ltd., Richmond BC), which resulted in an average slaughter age of 494 ± 3, 518 ± 4, and 457 ± 4 days for Angus, Charolais, and KC, respectively. The liver sample of each animal was collected immediately after slaughter and the tissue was dissected from approximately the same location on the right lobe with the fibrous capsule removed. Samples were separately bagged and labelled, and were immediately flash frozen in liquid nitrogen, transported on dry ice, and stored at −80 °C until RNA extraction.

### RNA isolation and purification

From the frozen liver samples, a total of 36 samples (12 from each breed) consisting of six samples from animals with extreme high and six extreme low RFI phenotypes from each of the three breeds were selected for total RNA extraction and consequently differential gene expression analyses. The frozen liver tissue of each steer was pulverised into fine powder using liquid nitrogen with a pre-chilled mortar and pestle on dry ice. Total RNA was then extracted from 10 mg of the pulverised tissue using a Qiagen RNeasy Plus Universal Kit (Qiagen, Toronto, ON, Canada) and further purified using a Zymo RNA Clean & Concentrator (Zymo, Irvine, CA, USA). RNA was quantified using a NanoDrop 2000 Spectrophotometer (Thermo Scientific, Wilmington, DE, USA) and was deemed acceptable if its absorbance (A260/280) was between 1.8 and 2.0. RNA integrity was confirmed using a TapeStation-Agilent instrument (Agilent Technologies, Mississauga, ON, Canada), and the RNA integrity number (RIN) values for all samples were higher than 8.

### cDNA library preparation and sequencing

Preparation of cDNA library and sequencing for each of the 36 animal samples were performed at the Clinical Genomics Centre (Toronto, ON, Canada), where mRNA was purified and enriched from 1 µg of each of the total RNA samples and then fragmented. Thereafter, the first strand of the cDNA was synthesized using SuperScript II cDNA kit (Thermo Fisher Scientific, San Jose, CA, USA) and the second strand was synthesized using the Illumina TruSeq^®^ RNA Sample Prep Kit v2 (Illumina, San Diego, CA, USA). The cDNA libraries were validated using gel electrophoresis to confirm that the fragment size was 150 bp (on average) and concentration was on average 25 ng/µl per sample. Unique oligonucleotide adapters were added to the cDNA of each sample to allow for multiplexing. Of the prepared sample cDNA libraries, 27 (all Angus, all KC and 3 Charolais samples) were single end sequenced (100 bp) under the high output run mode of the Illumina Hiseq 2500 System on eight flow cell lanes, while the other 9 Charolais samples were sequenced under the rapid run mode of the same sequencing equipment. High quality single end reads of 101 bp with an average Phred score of 36 and 37 for high output run mode and rapid run mode, respectively, were obtained with an average of 31 and 46 million reads per sample for high output run mode and rapid run mode, respectively. All sequence data generated for this study has been submitted to the Gene Expression Omnibus repository under the accession number GSE107477.

### RNA-seq data analyses

Raw single-end sequence reads for each sample were assessed for sequencing quality using FastQC (Version 0.11.5) with default parameters^58^. Reads of each sample were aligned and mapped to the bovine genome UMD3.1 using the TopHat (version 2.1.1) RNA-seq mapper with default single end read alignment parameters^59^. Reads that were uniquely aligned to each gene annotated in the GTF Bovine gene annotation file (ftp://ftp.ensembl.org/pub/release-89/gtf/bos_taurus/Bos_taurus.UMD3.1.89.gtf.gz) were counted using HTSeq-count with default parameters^60^ which generated the read count tables that were used for downstream differential gene expression statistical analyses.

### Differential gene expression statistical analysis

Gene read count tables from HTSeq-count, the annotation file downloaded from Ensembl Biomart (http://www.ensembl.org/biomart/martview/9153354bb2bef3f0fe8126460f4804ae), and sample information file were used for differential gene expression statistical analyses using edgeR^61^. Genes within each breed with less than one count per million (CPM) of mapped reads in at least six samples (half of the analyzed samples) were removed from further analyses as proposed by Anders *et al*.^62^. For the retained genes, their counts were normalized using the trimmed mean M values (TMM) method to account for the variation in library sequencing depths between samples^62^. The TMM normalization method implemented in edgeR was proposed by Robinson & Oshlack^63^, and it assumes that the majority of the sequenced genes in the libraries are not differentially expressed. With one sample considered as a reference, a TMM factor was calculated for each sample as a weighted mean of log ratios of gene-wise log fold changes and absolute expression level after exclusion of genes with the highest (30%) log-fold change ratios and highest (5%) absolute expression. The TMM value for each sample was expected to be equal or close to 1, if not, correction factors were calculated and applied to the original library sizes to calculate new effective library sizes. Normalized read counts were then analyzed with a generalized linear model for each of the breed populations with an assumption of a negative binomial distribution of gene counts to identify differentially expressed genes, as implemented in egdeR. The statistical models used for analyses are as described below:

$${\rm{M}}{\rm{o}}{\rm{d}}{\rm{e}}{\rm{l}}.1:\,{\rm{l}}{\rm{o}}{\rm{g}}\,{({\rm{C}}{\rm{P}}{\rm{M}})}_{{\rm{i}}{\rm{j}}{\rm{k}}{\rm{l}}}=\mu +{{\rm{R}}{\rm{F}}{\rm{I}}}_{{\rm{i}}}+{{\rm{S}}{\rm{I}}{\rm{R}}{\rm{E}}}_{{\rm{j}}}+{{\rm{e}}}_{{\rm{i}}{\rm{j}}{\rm{k}}{\rm{l}}}$$

$${\rm{M}}{\rm{o}}{\rm{d}}{\rm{e}}{\rm{l}}.2:\,{\rm{l}}{\rm{o}}{\rm{g}}\,{({\rm{C}}{\rm{P}}{\rm{M}})}_{{\rm{i}}{\rm{j}}{\rm{m}}{\rm{k}}{\rm{l}}}=\mu +{{\rm{R}}{\rm{F}}{\rm{I}}}_{{\rm{i}}}+{{\rm{S}}{\rm{I}}{\rm{R}}{\rm{E}}}_{{\rm{j}}}+{{\rm{S}}{\rm{E}}{\rm{Q}}}_{{\rm{m}}}+{{\rm{e}}}_{{\rm{i}}{\rm{j}}{\rm{m}}{\rm{k}}{\rm{l}}}$$

Model.1 was used for Angus and KC steer gene expression analyses, where log(CPM)_ijkl_ was the log transformed read counts per million of mapped reads for the gene l in sample k from i^th^ RFI group (high or low) and j^th^ SIRE group, and e_ijkl_ as the random error term. Model.2 was used for Charolais steer gene expression analyses, where log(CPM)_ijmkl_ was the log transformed counts per million of mapped reads for gene l in sample k from the i^th^ RFI group, j^th^ SIRE group, and m^th^ SEQ (sequencing mode), and e_ijmkl_ was the random error term. The term µ was the expected (average) gene expression in the breed population and RFI, SIRE and SEQ were treated as fixed effects in the models. For each model, the RFI group consisted of 6 steers with high RFI values in the high-RFI group and 6 steers with low RFI values in the low-RFI group. The SIRE effect of Angus, Charolais, and KC steers included 6, 5, and 9 sires, respectively. For Charolais, SEQ was included as an additional fixed effect to account for differences due to the sequencing modes (i.e. high output run mode or rapid run mode) (Model.2). Differentially expressed (DE) genes were identified using a likelihood ratio test of each gene expression level between the two RFI groups with the high-RFI group (or less feed efficient group) used as the reference group. The analysis was performed for each gene, therefore, Benjamin-Hochberg method was used to control the false discovery rate (FDR) due to multiple testing^64^. A threshold FDR of 0.05 and fold change (FC) of greater than two (>2) were used as the cut off to indicate significant differential gene expression.

### Functional enrichment analysis

To understand the biological functionality of the DE genes identified, functional analyses for the DE genes within each breed were performed using Ingenuity Pathway Analysis software (IPA) (Redwood City, CA; www.qiagen.com/ingenuity). Ensembl bovine gene IDs and log_2_-fold change (logFC) of the DE genes were used as identity (ID) and expression level (Observation 1), respectively, in IPA. To increase the number of mapped genes, Ensembl IDs for the unmapped genes were extracted and replaced with their closest human orthologue gene Ensembl IDs. Thereafter a combined list of bovine Ensembl for the mapped and human ortholog Ensembl IDs for unmapped genes was used for IPA biological function analysis. Molecular and cellular functions or biological functions were considered significantly enriched if the p-value for the overlap comparison test between the input gene list and the IPA Knowledge base database for a given biological function was less than 0.05. Activation or deactivation level of a specific enriched metabolic process within a biological function was defined by the Z-score that was calculated from the expression levels of the overlapping DE genes, where a negative or a positive score indicated deactivation or activation, respectively.

## Electronic supplementary material

S1

S2

S3

S4

S5
