# Supplementary material for: Transcriptome analyses reveal reduced hepatic lipid synthesis and accumulation in more feed efficient beef cattle
Source: Sci Rep. 2018 May 8;8:7303. doi: 10.1038/s41598-018-25605-3 (PMC5940658; doi:10.1038/s41598-018-25605-3)
Supplement: Supplementary file 5 — S5 [file 41598_2018_25605_MOESM5_ESM.doc]

**Transcriptome analyses reveal reduced hepatic lipid synthesis and accumulation in more feed efficient beef cattle**

Robert Mukiibi^1^, Michael Vinsky^2^, Kate A. Keogh^3^, Carolyn Fitzsimmons^1,2^, Paul Stothard^1^, Sinéad M. Waters^3^, & Changxi Li^1,2^

^1^Department of Agricultural, Food and Nutritional Science, University of Alberta, Edmonton, Alberta, Canada, T6G 2P5. ^2^Lacombe Research and Development Centre, Lacombe, Agriculture and Agri-Food Canada, Alberta, Canada, T4L 1W1, ^3^Animal and Bioscience Research Department, Teagasc, Grange, Dunsany, County Meath, Ireland.

Correspondence and requests for materials should be addressed to Dr. Changxi Li (email: [changxi.li@agr.gc.ca](mailto:changxi.li@agr.gc.ca) ).

S5- Enriched molecular and cellular functions


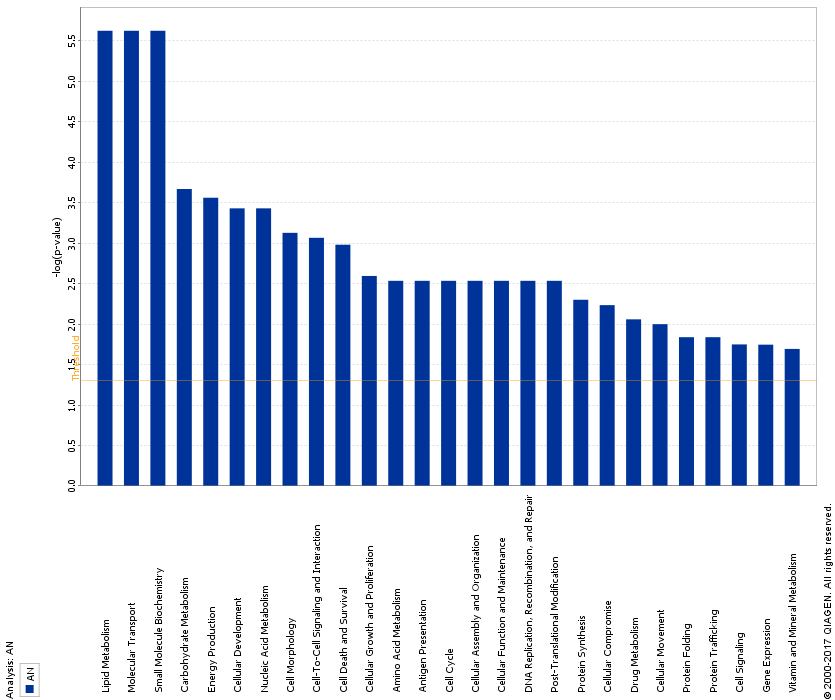


**Supplementary Figure 1. Enriched molecular and cellular functions for Angus**


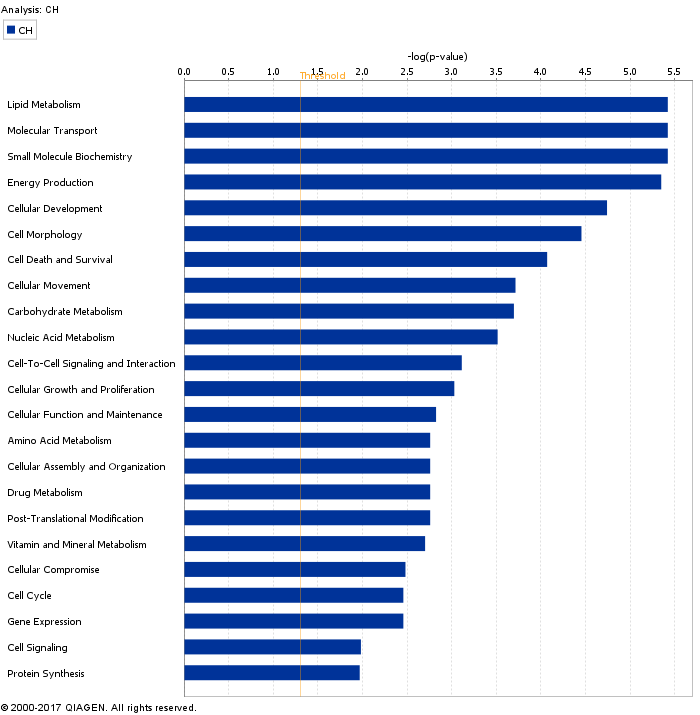


**Supplementary Figure 2. Enriched molecular and cellular functions for Charolais**


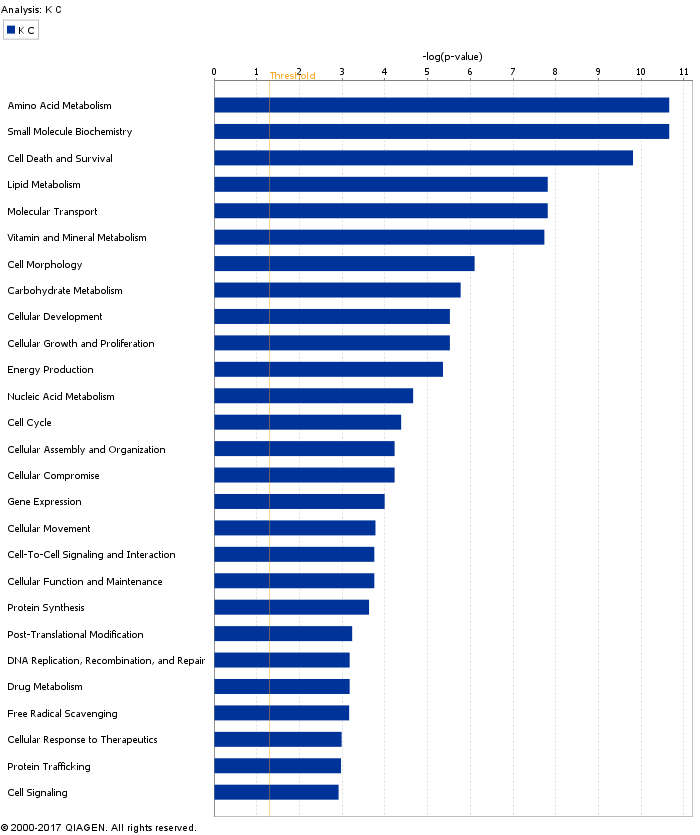


**Supplementary Figure 3. Enriched molecular and cellular functions for KC**
